# On the angular anisotropy of the randomly averaged magnetic neutron scattering cross section of nanoparticles

**DOI:** 10.1107/S205225252300180X

**Published:** 2023-03-14

**Authors:** Michael P. Adams, Evelyn Pratami Sinaga, Andreas Michels

**Affiliations:** a Department of Physics and Materials Science, University of Luxembourg, 162A avenue de la Faiencerie, L-1511 Luxembourg, Grand Duchy of Luxembourg; Lund University, Sweden; Keele University, United Kingdom

**Keywords:** nanoscience, magnetic scattering, computational modeling, nanostructure, neutron scattering, angular anisotropy, Stoner–Wohlfarth particles

## Abstract

The magnetic small-angle neutron scattering (SANS) cross section of dilute ensembles of uniformly magnetized and randomly oriented Stoner–Wohlfarth particles is calculated using the Landau–Lifshitz equation. The focus of this study is on the angular anisotropy of the magnetic SANS signal as it can be seen on a two-dimensional position-sensitive detector. Depending on the symmetry of the magnetic anisotropy of the particles (*e.g.* uniaxial, cubic), an anisotropic magnetic SANS pattern may result, even in the remanent state or at the coercive field. The case of inhomogeneously magnetized particles and the effects of a particle-size distribution and interparticle correlations are also discussed.

## Abstract 2

A discussion on the angular anisotropy of the two-dimensional magnetic small-angle neutron scattering cross section of randomly oriented nanoparticles is provided, and it is shown by means of micromagnetic simulations that this quantity is, in general, anisotropic.

## 1. Introduction

In a magnetic small-angle neutron scattering (SANS) experiment, the angular intensity distribution of the scattered neutrons on the two-dimensional position-sensitive detector usually provides the first information on the magnetic microstructure of the sample under study. When an external magnetic field **B** is applied and varied during the experiment, such images can yield useful information on the degree of magnetic saturation (at large fields), on the presence of clover-leaf-shaped angular anisotropies (at intermediate fields), or whether or not the magnetic moments are randomly distributed (at remanence or at the coercive field). The angular anisotropy of the magnetic SANS cross section can have many origins (Michels, 2021 ▸), *e.g.* (*a*) it can be due to the dipolar nature of the interaction between the magnetic moment of the neutron and the magnetic moment that is formed by the unpaired electrons of the sample, (*b*) it might be related to the magnetic interactions within the sample such as the magnetodipolar energy between magnetic moments, anisotropic exchange interaction or magnetic anisotropy, or (*c*) it can be due to the presence of a texture in the microstructure of the material.

One of the simplest examples is an ideal Langevin superparamagnet, which, by definition, consists of randomly oriented noninteracting single-domain nanoparticles (macrospins) that are embedded in a rigid nonmagnetic matrix. The magnetic behavior of this system is determined by the *B*/*T* ratio, where *T* is the absolute temperature. At remanence and not too low *T*, due to the randomizing effect of the thermal energy, the macrospins are randomly oriented in the matrix and the average system magnetization vanishes. The ensuing magnetic SANS cross section is then isotropically distributed in the detector plane. Applying a magnetic field induces an average magnetization, which may result in the appearance of an angular anisotropy of the scattering pattern [see the discussion in Section 5.1.2 of Michels (2021 ▸)].

Here, we consider the case of a statistically isotropic dilute ensemble of identical magnetic nanoparticles, so that case (*c*) is excluded as a source of the scattering anisotropy. Temperature effects are not taken into account. The particles are assumed to be in a single-domain state during the magnetization-reversal process, which implies that the only sample-related angular anisotropy that eventually becomes visible on the detector is due to magnetic anisotropy. As we will see below, cases (*a*) and (*b*) can be disentangled from one another. The magnetic nanoparticle ensemble is treated within the well known Stoner–Wohlfarth model (Stoner & Wohlfarth, 1948 ▸), which is a workhorse in magnetism, since it is the simplest approach for producing hysteresis effects. The Stoner–Wohlfarth model considers a system of noninteracting single-domain particles in the presence of an applied magnetic field. The particles exhibit magnetic anisotropy, which may have its origin in dipolar shape anisotropy and/or in spin-orbit-interaction-related magnetocrystalline anisotropy. We analyze the role played by the magnetic anisotropy for the angular anisotropy of the two-dimensional magnetic SANS cross section of Stoner–Wohlfarth particles. The Landau–Lifshitz (LL) equation of motion for the magnetization is employed to determine the magnetic equilibrium state and to calculate the corresponding magnetic SANS signal and the pair-distance distribution function.

This article is organized as follows. Section 2 displays the well known equations for the magnetic SANS cross section of a dilute ensemble of uniformly magnetized single-domain particles that are rigidly embedded in a nonmagnetic matrix. We consider the two most often used scattering geometries, which have the externally applied magnetic field either perpendicular or parallel to the incoming neutron beam. Section 3 briefly recapitulates the basic expressions of the Stoner–Wohlfarth model, while Section 4 presents and discusses the results for the SANS observables. We comment on the case of inhomogeneously magnetized particles and on the effect of interparticle correlations (dense packing). Finally, Section 5 summarizes the main findings of this work. In the supporting information of this article, we provide several movies that feature the average magnetization, the two- and one-dimensional magnetic SANS cross sections, as well as the pair-distance distribution function, correlation function, and anisotropy parameter during the magnetization-reversal process assuming different magnetic anisotropy symmetries (compare the six cases in Table 1).

## 2. Magnetic SANS cross section of a dilute ensemble of single-domain particles

**Figure 1:**
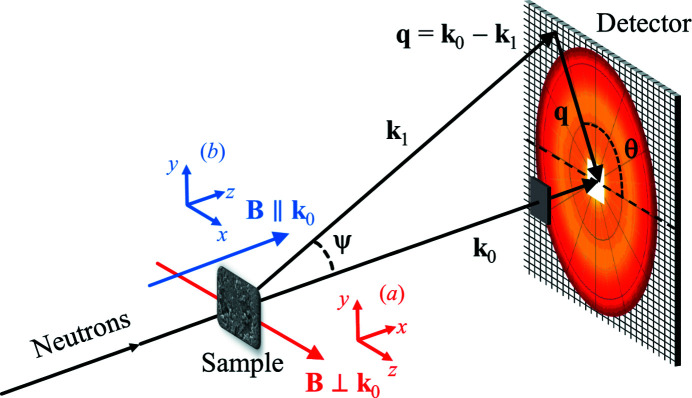
The two most often employed scattering geometries in magnetic SANS experiments. (*a*) External magnetic field **B** perpendicular to the incoming neutron beam (**B** ⊥ **k**_0_) and (*b*) **B** ∥ **k**_0_. Note that **B** ∥ **e**_*z*_ in both geometries. The momentum-transfer or scattering vector **q** corresponds to the difference between the wavevectors of the incident (**k**_0_) and scattered (**k**_1_) neutrons, *i.e.* **q** = **k**_0_ − **k**_1_; its magnitude for elastic scattering is given by 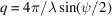 , where λ is the mean wavelength of the neutrons and ψ is the scattering angle. The angle θ is used to describe the angular anisotropy of the recorded scattering pattern on the two-dimensional position-sensitive detector.

Magnetic SANS experiments are usually conducted with the external magnetic field **B** either applied perpendicular (⊥) or parallel (∥) to the wavevector **k**_0_ of the incoming neutron beam [compare Figs. 1 ▸(*a*) and 1 ▸(*b*)]. For these two scattering geometries, the macroscopic elastic magnetic SANS cross section dΣ_*M*_/dΩ at momentum-transfer or scattering vector **q** can be expressed as (Michels, 2021 ▸) 

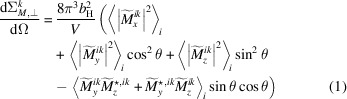

and 

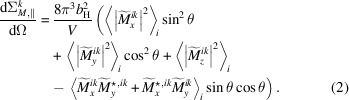

In these equations, *V* is the scattering volume, *b*_H_ = 2.91 × 10^8^ A^−1^m^−1^ denotes the magnetic scattering length in the small-angle regime, 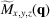 are the Cartesian Fourier components of the magnetization vector field **M**(**r**) = [*M*_*x*_(**r**), *M*_*y*_(**r**), *M*_*z*_(**r**)], the index *i* refers to the orientation of particle *i*, the index *k* keeps track of the applied-field value, the asterisk 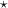 denotes the complex-conjugated quantity and the 〈...〉_*i*_ notation is explained below in equation (7). In the perpendicular and parallel scattering geometries, the scattering vector is given by 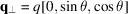 and 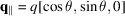 , where the angle θ is measured between **q**_⊥_ and **B** ∥ **e**_*z*_ and **q**_∥_ and **e**_*x*_, respectively. Note that **B** ∥ **e**_*z*_ in both geometries. Equations (1) and (2) neglect interparticle interference effects and are valid for a dilute scattering system.

In the main part of this article we exclusively focus on the perpendicular scattering geometry, and we refer to the supporting information for movies that show results for the parallel geometry.

The Fourier transform of the magnetization vector field of a nanoparticle is defined by 

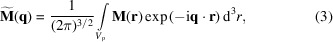

which, for a uniformly magnetized particle, can be simplified to 

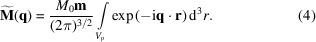

In going from equation (3) to equation (4), we have expressed the (constant) magnetization vector as **M** = *M*_0_ **m**, where *M*_0_ is the saturation magnetization and **m** is a unit vector along **M**. The remaining integral in equation (4) is the well known form-factor integral (over the volume *V*_p_ of the particle), which is analytically known for many particle shapes. For spherical particles (with radius *R*), equation (4) can be further simplified to

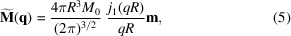

where *j*_1_(*z*) denotes the first-order spherical Bessel function. For more complicated particle shapes (*e.g.* cylinders or flat discs), an additional average over the particle orientation might be required to obtain the only *q*-dependent form factor. As a reminder, we consider a dilute system of *N* identical spherical single-domain particles, where, at a given value of the applied field *B* ^*k*^, each particle *i* has its own random orientation of magnetic easy axes with respect to **B** (to be further specified in Section 3). For this situation, we may express the Fourier components as 

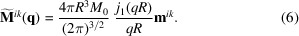

The magnetic SANS cross sections at the *k*th magnetic field value, averaged over all the random easy-axis orientations *i*, are then given by equations (1) and (2), where the bracket notation of the mean operator is defined as follows: 

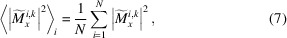

and similar for the other Fourier components. Since the spherical Bessel function is a scalar prefactor to the Fourier transform of the magnetization vector, we can simplify the magnetic SANS cross sections as follows: 

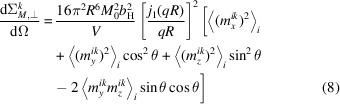

and 

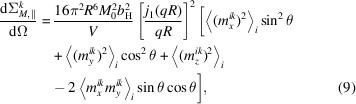

such that the magnetic SANS cross section directly follows from the (real valued) real-space correlation functions of the components of the magnetization vector. We therefore define the cross-correlation matrix corresponding to the *k*th value of the magnetic field as 

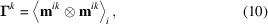

where more explicitly written the components are defined as 

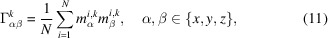

such that the magnetic SANS cross sections are written as 

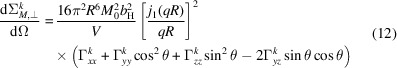

and 

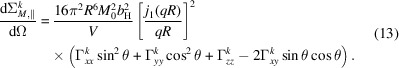

In the general case of an inhomogeneous magnetization distribution, *i.e.* **m** = **m**(**r**), these formulations of the magnetic SANS cross sections only correspond to the first term in a Taylor series expansion. From equations (12) and (13) we see that the magnetic SANS cross section gives quite different insights into the magnetization structure than the hysteresis loop: the latter contains information about the first-order moments, while the former yields information about the second-order moments of the magnetization vector field. The magnetic SANS cross section of uniformly magnetized particles is anisotropic (θ dependent) when the terms in the brackets on the second lines of equations (12) and (13) add up to yield a resulting net θ dependence. This statement can be further specified by noting the symmetry of the equations in the parallel scattering geometry [equation (13)], which is absent in the perpendicular case [equation (12)]. For **B** ∥ **k**_0_ the two transversal magnetization components lie in the detector plane, whereas for **B** ⊥ **k**_0_ only one transversal component lies in the detector plane and the other one is pointing along the incident-beam direction [compare Figs. 1 ▸(*a*) and 1 ▸(*b*)]. Since for the here-considered Stoner–Wohlfarth system with **B** ∥ **e**_*z*_ in both scattering geometries we have Γ_*xx*_ = Γ_*yy*_ and Γ_*xy*_ = Γ_*xz*_ = Γ_*yz*_ = 0 (see Appendix *A* [App appa]), it becomes immediately clear that the two-dimensional dΣ_*M*, ∥_/dΩ is isotropic at all fields, while dΣ_*M*, ⊥_/dΩ is generally anisotropic. Moreover, for a constant magnetization vector field, the orientationally averaged form-factor integral in equation (4) can be analytically or numerically computed for many particle shapes, with the result that the prefactor in equations (12) and (13) is only a function of the magnitude of **q**. Therefore, the above result – isotropy of dΣ_*M*, ∥_/dΩ and general anisotropy of dΣ_*M*, ⊥_/dΩ – is true for arbitrary particle shapes and also in the presence of a distribution of particle sizes, as long as all the randomly oriented particles are in a single-domain state.

The azimuthally averaged SANS cross sections are straightforwardly obtained as 

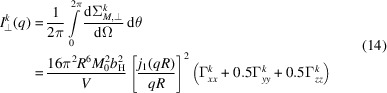

and 

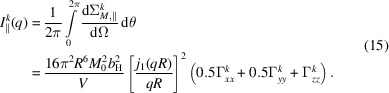

Here, for the 2π azimuthal average, we see that the *yz* and *xy* cross-correlation terms vanish, and only the autocorrelation terms remain. The pair-distance distribution functions are obtained as 

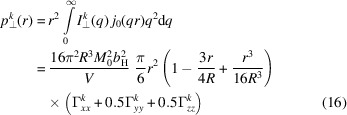

and 

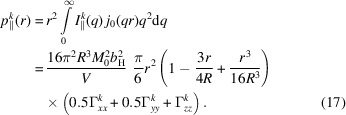

The related correlation functions *c* ^*k*^(*r*) = *p* ^*k*^(*r*)/*r* ^2^ are 

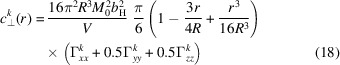

and 

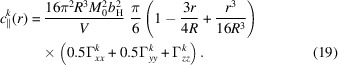

The 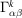 are real numbers, which depend on the applied field and on the symmetry of the magnetic anisotropy of the particles. We then see that – within the present Stoner–Wohlfarth approach – the *I*(*q*), *p*(*r*) and *c*(*r*) are identical for the perpendicular and parallel scattering geometries, except for a numerical prefactor.

To quantify the angular anisotropy of the two-dimensional magnetic SANS cross section, we introduce (for **B** ⊥ **k**_0_) the following number [compare Fig. 1 ▸ with equation (12)]: 

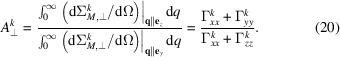

For the parallel scattering geometry, where **B** is perpendicular to the detector plane, we calculate *A* ^*k*^ similar to equation (20) as the ratio of integrated intensities along the horizontal and vertical directions on the detector [compare with equation (13)]: 

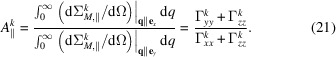

In the following, the quantities *A*_⊥_ and *A*_∥_ are denoted as the anisotropy parameters. As discussed before, since Γ_*xx*_ = Γ_*yy*_, we find that *A*_∥_ = 1 at all fields, while generally *A*_⊥_ ≠ 1 (see Appendix *A* [App appa]). When the Γ_*zz*_ correlation coefficient dominates, we have *A*_⊥_ < 1 and the two-dimensional magnetic SANS signal exhibits a dominant 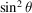 anisotropy, whereas for dominant Γ_*xx*_ and Γ_*yy*_ (*A*_⊥_ > 1) we observe a 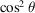 type angular anisotropy.

## 3. Recap: Stoner–Wohlfarth model

In this chapter, we recapitulate the basic ideas of the Stoner–Wohlfarth model (Stoner & Wohlfarth, 1948 ▸), which considers a magnetically anisotropic single-domain particle in the presence of an applied magnetic field **B** (assumed here to be parallel to the *z* direction of a Cartesian laboratory coordinate system). The origin of the magnetic anisotropy can be due to shape anisotropy and/or magnetocrystalline anisotropy. Here, we consider identical particles possessing magnetocrystalline anisotropy only. Note also that spherical particles do not exhibit shape anisotropy and thermal effects are ignored. By denoting with 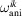 the magnetic anisotropy energy density in the (global) laboratory coordinate system, the total energy density ω^*ik*^ of a particle *i* at field *k* is commonly expressed as 

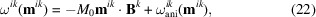

where *M*_0_ is the saturation magnetization of the material. The two most common forms of magnetocrystalline anisotropy either exhibit uniaxial (u) or cubic (c) symmetry. The corresponding mathematical expressions for the magnetic anisotropy energy densities, in the local coordinate frame of the particle, are the following: 

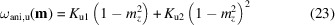

and 

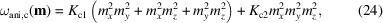

where *K*_u_ and *K*_c_ are the temperature-dependent anisotropy constants (in J m^−3^). Depending on their relative magnitude and the signs of the anisotropy constants, different easy axes are obtained (Kronmüller & Fähnle, 2003 ▸). The corresponding effective magnetic fields (in Tesla) are then readily obtained as 

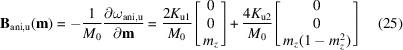

and 

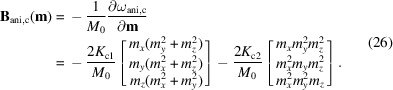

The different (random) particle orientations *i* are obtained by rotations in three-dimensional space (change of basis). This is accomplished by using a rotation matrix **R**_*i*_ that is parametrized by (random) Euler angles γ_*i*_, δ_*i*_ and ε_*i*_ (with 0 ≤ γ_*i*_ ≤ 2π, 0 ≤ δ_*i*_ ≤ π and 0 ≤ ε_*i*_ ≤ 2π) (Goldstein, 1991 ▸), so that the total effective field is calculated as follows: 

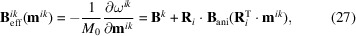

where 

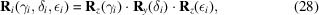

and the superscript T refers to the transpose of the matrix. Here we adopt a *z*–*y*–*z* rotation sequence. The procedure of obtaining the Euler angles starts with uniformly distributed random numbers *a*_*i*_, *b*_*i*_ and *c*_*i*_ in the three-dimensional unit cube, such that 0 ≤ *a*_*i*_, *b*_*i*_, *c*_*i*_ ≤ 1. As a random-number generator we use the low-discrepancy Sobol sequences (https://www.mathworks.com/help/stats/sobolset.html). In order to achieve a uniform distribution of random angles on the unit sphere, we use the following transformations: 

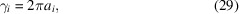




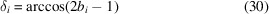

and 

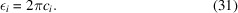

To obtain the static equilibrium magnetization, we insert the expression for 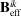 into the LL equation (Bertotti, 1998 ▸), which describes the magnetization dynamics: 

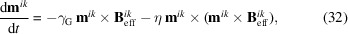

where γ_G_ = 1.76 × 10^11^ T^−1^s^−1^ is the gyromagnetic ratio and η is the damping constant. Following the temporal evolution of the LL equation, the static spin structure 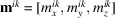 of nanomagnet *i* at field *k* can be obtained. Repeating (at fixed *k*) these simulations *N* times for different easy-axis orientations allows us to compute the averages that determine the magnetic SANS cross section. More specifically, the hysteresis loop of the ensemble of spherical nanomagnets then follows from the averaged magnetization projected along the *z* direction, 

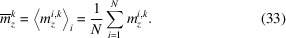

In addition to 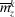 , we also calculate the field dependence of the transversal magnetization components, 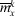 and 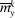 , as well as the field loops of the components of the cross-correlation matrix Γ_αβ_ (with α, β ∈ {*x*, *y*, *z*}) [equation (11)]. As we have seen in Section 2, these are of particular relevance for the magnetic SANS cross section.

In the numerical computations, we used the following parameters: η = 3 × 10^11^ T^−1^s^−1^ and an integration time step of 5 × 10^−15^ s. Typically, *K* = 2000 discretization points for the applied magnetic field and *N* = 10 000 samples of different orientations for the easy axes of the particles (angles γ_*i*_, δ_*i*_ and ε_*i*_) were used. For further details on the SANS simulation methodology using the LL equation, we refer to Adams *et al.* (2022*a* ▸).

## 4. Results and discussion

In our analysis, we consider the following six cases for Stoner–Wohlfarth particles with uniaxial and cubic anisotropy: (i) *k*_u1_ = +1 and *k*_u2_ = 0, (ii) *k*_u1_ = −1 and *k*_u2_ = 0, (iii) *k*_u1_ = −0.5 and *k*_u2_ = +0.5, (iv) *k*_c1_ = +1 and *k*_c2_ = 0, (v) *k*_c1_ = −1 and *k*_c2_ = 0, and (vi) *k*_c1_ = −1 and *k*_c2_ = +9. The *k*_u, c_ (in Tesla) are related to the *K*_u, c_ (in J m^−3^) via *k*_u, c_ = 2*K*_u, c_/*M*_0_ [compare equations (25) and (26)]. Minimization of the anisotropy energy densities [equations (23) and (24)] shows that these combinations of anisotropy constants correspond to the following well known easy-axis orientations in hexagonal and cubic single crystals (Kronmüller & Fähnle, 2003 ▸): (i) easy *c* axis, (ii) easy basal plane, (iii) easy cone with opening angle 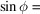 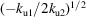 , (iv) 〈100〉 directions, (v) 〈111〉 directions and (vi) 〈110〉 directions.

**Figure 2:**
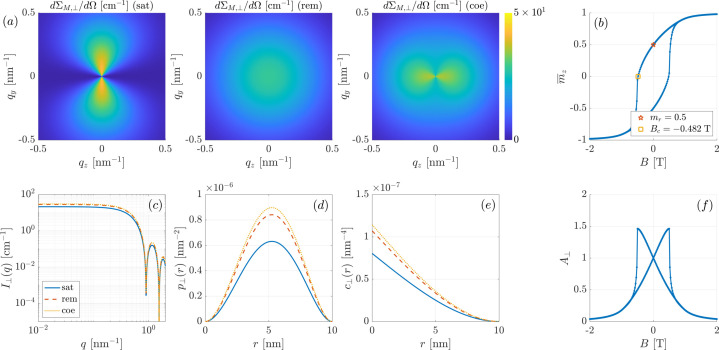
Results for the magnetization and the SANS observables of a dilute ensemble of uniaxial Stoner–Wohlfarth particles with *k*_u1_ = +1 and *k*_u2_ = 0 [case (i)] (**B** ⊥ **k**_0_). The (spherical) particle diameter is *D* = 10 nm. (*a*) Two-dimensional magnetic SANS cross sections dΣ_*M*, ⊥_/dΩ at saturation (sat), remanence (rem) and at the coercive field (coe); (*b*) hysteresis loop 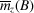 (the reduced remanence and the coercivity are indicated); (*c*) one-dimensional 2π azimuthally averaged magnetic SANS cross sections 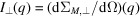 ; (*d*) pair-distance distribution functions *p*_⊥_(*r*); (*e*) correlation functions *c*_⊥_(*r*); and (*f*) anisotropy parameter *A*_⊥_(*B*).

Table 1 ▸ contains the values of the second moments Γ_αα_ of the components of the magnetization vectors at selected points on the hysteresis loop (remanence and coercivity), and for the different anisotropy symmetries. The hysteresis loops and the full field dependencies of the autocorrelations are shown in Appendix *A* [App appa]. As an example, for uniaxial Stoner–Wohlfarth particles of case (i), Fig. 2 ▸ depicts the results for the SANS observables. Inspection of the table entries for the Γ_αα_ and comparison with the magnetic SANS cross sections [equations (12) and (13)] reveals that only case (i) yields, in the perpendicular scattering geometry, an isotropic two-dimensional SANS image at remanence. In all other cases, we find (for **B** ⊥ **k**_0_) an anisotropic magnetic SANS pattern at remanence and at the coercive field. By contrast, in the parallel scattering geometry (**B** ∥ **k**_0_), we observe (since Γ_*xx*_ = Γ_*yy*_) an isotropic magnetic dΣ_*M*, ∥_/dΩ at all fields during the magnetization-reversal process.

Considering case (i), we see that the angular anisotropy of the two-dimensional dΣ_*M*, ⊥_/dΩ changes strongly between saturation ( 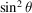 type), remanence (isotropic) and the coercive field ( 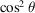 type) [Fig. 2 ▸(*a*)], while the azimuthally averaged dΣ_*M*, ⊥_/dΩ changes relatively little between these situations [Fig. 2 ▸(*c*)]. Decreasing the field from saturation (where Γ_*xx*_ = Γ_*yy*_ = 0 and Γ_*zz*_ = 1) to zero field and to *B*_*c*_, we observe an increase of the one-dimensional dΣ_*M*, ⊥_/dΩ and of the pair-distance distribution function *p*_⊥_(*r*) [Fig. 2 ▸(*d*)] and of the correlation function *c*_⊥_(*r*) [Fig. 2 ▸(*e*)] [compare equations (16) to (19)]. From these results it may be concluded that, depending on the anisotropy symmetry of Stoner–Wohlfarth particles, an anisotropic magnetic SANS pattern is (generally) obtained; see Fig. 2 ▸(*f*) for the anisotropy parameter *A*_⊥_(*B*). In experimental studies, where often the two-dimensional total (nuclear and magnetic) dΣ_⊥_/dΩ is analyzed, one should therefore be cautious in assuming that an isotropic pattern is to be expected at characteristic field values such as at remanence or at the coercive field. To access the purely magnetic SANS cross section in unpolarized experiments, the subtraction of the total dΣ_⊥_/dΩ at a field close to magnetic saturation from the data at lower fields might help. As shown in the work by Bersweiler *et al.* (2019 ▸) on Mn—Zn ferrite nanoparticles, an isotropic total dΣ_⊥_/dΩ at zero field can then turn into an anisotropic purely magnetic signal, in this way providing access to the magnetic correlations. Of course, polarization analysis also yields the purely magnetic SANS cross section, albeit with much more effort regarding the experimental setup and the data-reduction procedure.

We refer to the supporting information of this article, where several movies that feature the average magnetization 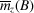 and the SANS observables during the magnetization-reversal process are provided. More specifically, for the six cases specified in Table 1 ▸, we display, for both scattering geometries, the two- and one-dimensional magnetic SANS cross sections dΣ_*M*, ⊥_/dΩ and dΣ_*M*, ∥_/dΩ, the pair-distance distribution functions *p*_⊥_(*r*) and *p*_∥_(*r*), the correlation functions *c*_⊥_(*r*) and *c*_∥_(*r*), and the anisotropy parameters *A*_⊥_ and *A*_∥_.

As mentioned already in Section 2 when discussing equations (12) and (13), within the present Stoner–Wohlfarth approach, the presence of a particle-size distribution results in the smearing of the form-factor oscillations (*i.e.* affects the *q* dependence) but leaves the angular anisotropy of dΣ_*M*, ⊥_/dΩ unchanged.

So far the discussion has been based on uniformly magnetized Stoner–Wohlfarth particles. For nonuniformly magnetized nanoparticles, where the magnetization vector field **m** = **m**(**r**) is a function of the position **r** within the particle, the dΣ_*M*, ⊥_/dΩ at remanence or at the coercive field is generally also expected to depend on the angle θ in the detector plane. Nonuniformities in the magnetization distribution of nanoparticles are *e.g.* caused by surface anisotropy, vacancies or antiphase boundaries (Nedelkoski *et al.*, 2017 ▸; Ijiri *et al.*, 2019 ▸; Zákutná *et al.*, 2020 ▸; Lak *et al.*, 2021 ▸; Köhler *et al.*, 2021 ▸; Honecker *et al.*, 2022 ▸; Adams *et al.*, 2022*b* ▸,*a* ▸; Sinaga *et al.*, 2023 ▸). Micromagnetic simulations that take into account the relevant interactions such as isotropic exchange, antisymmetric exchange, magnetic anisotropy, Zeeman energy and the magnetodipolar interaction are an important tool for advancing the understanding of magnetic SANS of nanomagnets (Michels, 2021 ▸). Unfortunately, due to the nonlinearity of the underlying integro-differential equations of micromagnetics, numerical simulations have to be carried out.

**Figure 3:**
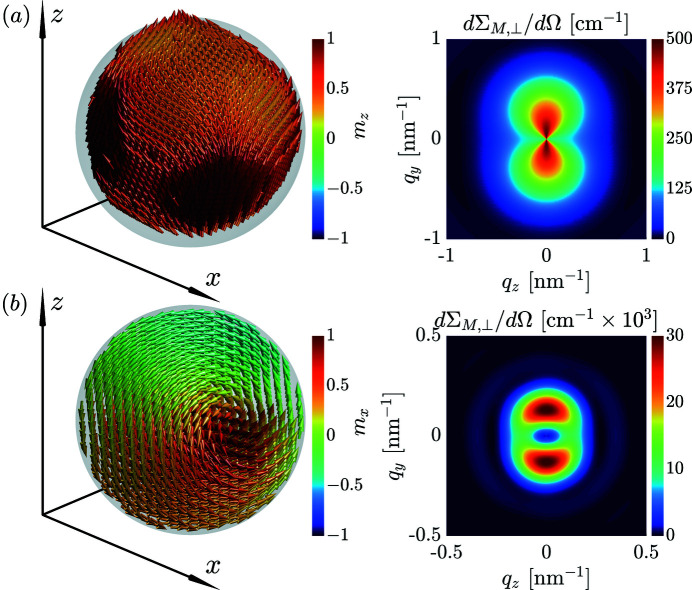
Micromagnetic simulation results for dilute ensembles of inhomogeneously magnetized nanoparticles. (*a*) Remanent spin structure of a spherical 8 nm-sized nanoparticle with strong Néel surface anisotropy and corresponding randomly averaged magnetic SANS cross section dΣ_*M*, ⊥_/dΩ (**B** ⊥ **k**_0_) (Adams *et al.*, 2022*a* ▸). (*b*) Remanent spin structure and randomly averaged dΣ_*M*, ⊥_/dΩ of spherical nanoparticles with a diameter of 32 nm (Sinaga *et al.*, 2023 ▸).

As an example, we show in Fig. 3 ▸ selected results that feature an anisotropic (randomly averaged) dΣ_*M*, ⊥_/dΩ at remanence; for details on the micromagnetic SANS simulation methodology see the works of Adams *et al.* (2022*a* ▸) and Sinaga *et al.* (2023 ▸). Fig. 3 ▸(*a*) showcases the results of atomistic SANS simulations, where the focus is set on the effect of the Néel surface anisotropy on the spin structure and ensuing magnetic SANS signal of randomly oriented nanoparticles. This particular form of surface anisotropy arises because in a nanomagnet a significant fraction of atoms belong to the surface (with no neighbors on one side), and their magnetic properties such as exchange and anisotropy can be strongly modified relative to the bulk atoms. The snapshot of the real-space spin structure clearly reveals a significant spin disorder in the near-surface region of the nanoparticle, with a corresponding characteristic 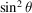 type anisotropic magnetic SANS pattern. Fig. 3 ▸(*b*) displays the results for a random ensemble of spherical nanoparticles. In this system (with no surface anisotropy), the magnetization distribution is determined by the dipolar interaction energy, which gives rise to a vortex-type spin texture. The randomly averaged dΣ_*M*, ⊥_/dΩ also exhibits a pronounced θ dependence.

The simulation results in Fig. 3 ▸ were obtained for a dilute set of nanoparticles, *i.e.* interparticle correlations are not taken into account. When the particle concentration in a sample increases, positional correlations become important, which is taken into account by the structure factor 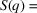 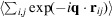 , where **r**_*ij*_ = **r**_*j*_ − **r**_*i*_ denotes the vector connecting the position vectors of particles *i* and *j*, and the bracket 〈...〉 refers to an orientational average. For magnetic particles, whether uniformly or nonuniformly magnetized, additional magnetic moment correlations become relevant, resulting in the appearance of a magnetic structure factor. This was realized by Honecker *et al.* (2020 ▸), who showed that the magnetic structure factor can deviate significantly from the nuclear (positional) structure factor for magnetically interacting nanoparticle ensembles; see also Hayter & Pynn (1982 ▸) and Pynn *et al.* (1983 ▸), where the structure factor of a magnetically saturated ferrofluid was derived. Since correlations between the particle magnetizations are magnetic field dependent and also anisotropic (Gazeau *et al.*, 2002 ▸; Honecker *et al.*, 2020 ▸), extending the present Stoner–Wohlfarth approach to higher concentrations (increased magnetodipolar interaction) does presumably not change the main statement of the present work, namely that the magnetic SANS cross section of a randomly oriented nanoparticle ensemble is, in the **B** ⊥ **k**_0_ geometry, generally anisotropic. This assertion is supported by the results of large-scale micromagnetic simulations on magnetic nanocomposites (Erokhin *et al.*, 2012 ▸; Michels *et al.*, 2014 ▸), which clearly show that the dipolar interaction results in an anisotropic magnetic SANS cross section in the perpendicular geometry.

## 5. Conclusions

We have analyzed the angular anisotropy of the magnetic SANS cross section of spherical Stoner–Wohlfarth particles using the Landau–Lifshitz equation. Depending on the symmetry of the magnetic anisotropy of the particles (uniaxial, cubic), an anisotropic randomly averaged magnetic SANS pattern may result in the perpendicular scattering geometry, even in the remanent or fully demagnetized state. The magnetic scattering in the parallel geometry is, as expected, isotropic. Inhomogeneously magnetized nanoparticles also generally exhibit an anisotropic randomly averaged magnetic SANS response. From the experimental point of view, the subtraction of the total unpolarized (nuclear and magnetic) scattering at saturation from data at lower fields might help to access the intrinsic anisotropy of the particles. Likewise, this can also be achieved by one-dimensional polarization analysis via the measurement of the spin-flip SANS cross section. Since the present Stoner–Wohlfarth simulations are relatively easy to implement, we recommend carrying them out in parallel to experimental investigations on magnetic nanoparticles. 

## Supplementary Material

Movie for case (i). DOI: 10.1107/S205225252300180X/fs5216sup1.mp4

Movie for case (ii). DOI: 10.1107/S205225252300180X/fs5216sup2.mp4

Movie for case (iii). DOI: 10.1107/S205225252300180X/fs5216sup3.mp4

Movie for case (iv). DOI: 10.1107/S205225252300180X/fs5216sup4.mp4

Movie for case (v). DOI: 10.1107/S205225252300180X/fs5216sup5.mp4

Movie for case (vi). DOI: 10.1107/S205225252300180X/fs5216sup6.mp4

## Figures and Tables

**Figure 4:**
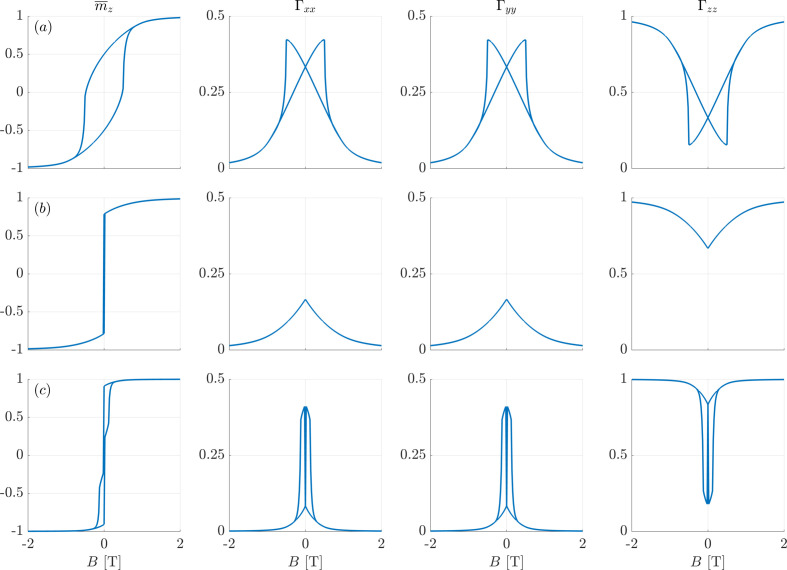
Hysteresis loops 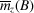 (the reduced remanences and coercivities are indicated) and second moments Γ_αα_ of the components of the magnetization vectors from the Stoner–Wohlfarth model with uniaxial anisotropy. (*a*) *k*_u1_ = +1 and *k*_u2_ = 0, (*b*) *k*_u1_ = −1 and *k*_u2_ = 0, and (*c*) *k*_u1_ = −0.5 and *k*_u2_ = +0.5.

**Figure 5:**
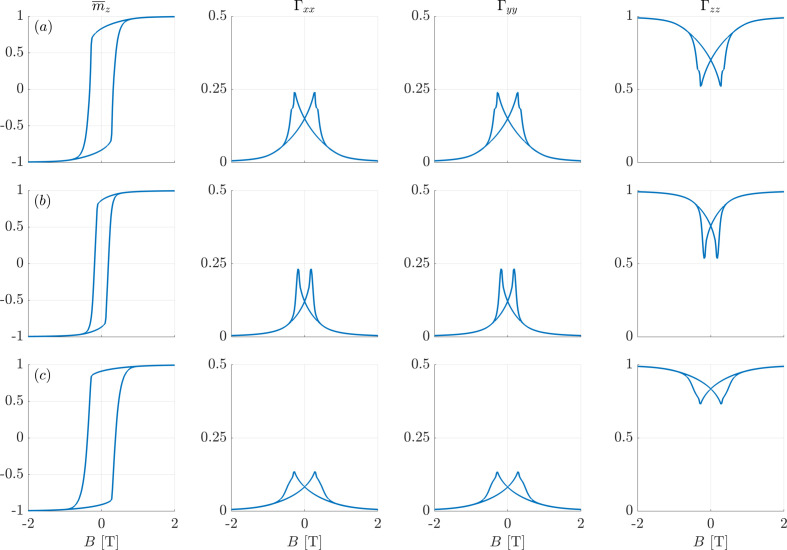
Similar to Fig. 4 ▸, but for cubic anisotropy. (*a*) *k*_c1_ = +1 and *k*_c2_ = 0, (*b*) *k*_c1_ = −1 and *k*_c2_ = 0, and (*c*) *k*_c1_ = −1 and *k*_c2_ = +9.

**Table 1:** Stoner–Wohlfarth particles with uniaxial and cubic anisotropy (Usov & Peschany, 1997 ▸) Values for the reduced remanence *m*_*r*_, the coercivity *B*_*c*_ (in Tesla), and for the autocorrelations Γ_*xx*_, Γ_*yy*_ and Γ_*zz*_ [equation (11)] at these points on the hysteresis loop. All cross-correlations Γ_αβ_ with α ≠ β vanish. The *k*_u, c_ are given in units of Tesla.

	*m*_*r*_	*B*_*c*_	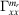	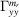	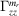	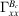	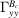	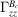
Case (i): uniaxial								
(*k*_u1_ = +1, *k*_u2_ = 0)	0.5	0.482	0.333	0.333	0.333	0.422	0.422	0.156
Case (ii): uniaxial								
(*k*_u1_ = −1, *k*_u2_ = 0)	0.785	0	0.167	0.167	0.667	–	–	–
Case (iii): uniaxial								
(*k*_u1_ = −0.5, *k*_u2_ = +0.5)	0.909	0	0.083	0.083	0.833	–	–	–
Case (iv): cubic								
(*k*_c1_ = +1, *k*_c2_ = 0)	0.831	0.321	0.150	0.150	0.700	0.1875	0.1875	0.625
Case (v): cubic								
(*k*_c1_ = −1, *k*_c2_ = 0)	0.866	0.189	0.121	0.121	0.758	0.225	0.225	0.550
Case (vi): cubic								
(*k*_c1_ = −1, *k*_c2_ = +9)	0.912	0.383	0.082	0.082	0.836	0.105	0.105	0.790

## Appendix A Magnetization curves and cross-correlation values of spherical Stoner–Wohlfarth particles with uniaxial and cubic anisotropy

Figs. 4 ▸ and 5 ▸ show the hysteresis loops  and the field dependencies of the second moments of the components of the magnetization vectors, Γ_αα_(*B*), from the Stoner–Wohlfarth model for uniaxial and cubic anisotropy. The six cases specified in Table 1 ▸ are considered. All cross-correlations Γ_αβ_ with α ≠ β vanish, and  .
